# Mucosal Healing Effectiveness and Safety of Anaprazole, a Novel PPI, vs. Rabeprazole in Patients With Duodenal Ulcers: A Randomized Double-Blinded Multicenter Phase II Clinical Trial

**DOI:** 10.3389/fmed.2021.690995

**Published:** 2021-07-14

**Authors:** Xu Shu, Zhenhua Zhu, Yu Fu, Zhenyu Zhang, Jiangbin Wang, Xing Li, Shuixiang He, Huizhen Fan, Side Liu, Guoxin Zhang, Jianhua Tang, Caibin Huang, Qin Du, Xiaoyan Wang, Baohong Xu, Yiqi Du, Qikui Chen, Bangmao Wang, Ying Chen, Xianghui Duan, Yong Xie, Lijuan Huo, Xiaohua Hou, Nonghua Lu

**Affiliations:** ^1^Department of Gastroenterology, The First Affiliated Hospital of Nanchang University, Nanchang, China; ^2^Department of Gastroenterology, Union Hospital, Tongji Medical College, Huazhong University of Science and Technology, Wuhan, China; ^3^Department of Gastroenterology, Nanjing First Hospital, Nanjing Medical University, Nanjing, China; ^4^Department of Gastroenterology, China–Japan Union Hospital of Jilin University, Changchun, China; ^5^Department of Gastroenterology, Jiangxi Pingxiang People's Hospital, Pingxiang, China; ^6^Department of Gastroenterology, First Affiliated Hospital of Xi'an Jiaotong University, Xi'an, China; ^7^Department of Gastroenterology, Yichun People's Hospital, Yichun, China; ^8^Department of Gastroenterology, Nanfang Hospital, Nanfang Medical University, Guangzhou, China; ^9^Department of Gastroenterology, Jiangsu Province Hospital, Nanjing, China; ^10^Department of Gastroenterology, Ganzhou People's Hospital, Ganzhou, China; ^11^Department of Gastroenterology, First Affiliated Hospital of Gannan Medical University, Ganzhou, China; ^12^Department of Gastroenterology, The Second Affiliated Hospital of Zhejiang University School of Medicine, Hangzhou, China; ^13^Department of Gastroenterology, The Third Hospital of Central South University, Changsha, China; ^14^Department of Gastroenterology, Beijing Luhe Hospital, Capital Medical University, Beijing, China; ^15^Department of Gastroenterology, Changhai Hospital, Shanghai, China; ^16^Department of Gastroenterology, Sun Yat-sen Memorial Hospital, Sun Yat-sen University, Guangzhou, China; ^17^Department of Gastroenterology, Tianjin Medical University General Hospital, Tianjin, China; ^18^Clinical Development, Xuanzhu Biopharmaceutical Co., Ltd., Beijing, China; ^19^Statistics, Xuanzhu Biopharmaceutical Co., Ltd., Beijing, China; ^20^Department of Gastroenterology, The First Hospital of Shanxi Medical University, Taiyuan, China

**Keywords:** proton pump inhibitors, duodenal ulcer, anaprazole, rabeprazole, peptic ulcer

## Abstract

**Background:** Proton pump inhibitors (PPIs) are validated gastric acid suppressors and have been widely used to treat patients with active duodenal ulcers. Although existing PPIs have shown great efficacy, many scientists are still devoted to developing more effective PPIs with better safety profile. Herein, we aimed to compare the safety and efficacy of anaprazole in duodenal mucosal healing, a novel PPI, to that of rabeprazole.

**Methods:** In this multicenter, randomized, positive-controlled, double-blinded, parallel-group phase II clinical trial, a total of 150 qualified patients with endoscopically confirmed active duodenal ulcers were randomized (1:1:1) to receive rabeprazole 10 mg, anaprazole 20 mg or anaprazole 40 mg for 4 weeks. The ulcer healing rates after 4 weeks of treatment were compared between groups by independent central review and investigator review. In addition, symptoms and safety were evaluated.

**Results:** Based on the independent central review, the ulcer healing rates of the 10 mg rabeprazole, 20 mg anaprazole and 40 mg anaprazole groups were 88.0, 85.1, and 87.5%, respectively, in the FAS population and 88.9, 86.0, and 90.9%, respectively, in the PPS population. The ulcer healing rate difference between anaprazole 20 mg and Rabeprazole 10 mg is −2.9% (95% CI, −16.5–10.7%), and −0.5% (95% CI, −13.5–12.5%) between anaprazole 40 mg and Rabeprazole 10 mg, in the FAS population. Based on the investigator review, the ulcer healing rates of the 10 mg rabeprazole, 20 mg anaprazole, and 40 mg anaprazole groups were 72.0, 70.2, and 77.1%, respectively, in the FAS population and 75.6, 72.1, and 79.5%, respectively, in the PPS population. The ulcer healing rate difference between anaprazole 20 mg and Rabeprazole 10 mg is −1.8% (95% CI, −19.8–16.3%), and 5.1% (95% CI, −12.2–22.3%) between anaprazole 40 mg and Rabeprazole 10 mg, in the FAS population. Most patients (>90%) eventually achieved complete symptom relief. The incidence rates of adverse events were of no significant differences among the treatment groups. Potential possible better liver tolerance was observed in two anaprazole dose groups than rabeprazole 10 mg group.

**Conclusion:** Both at a dosage of 20 and 40 mg daily, anaprazole, is effective with good safety profile in the treatment of active duodenal ulcers in this Phase 2 study, which allows anaprazole to be advanced to a phase III clinical trial.

**Clinical Trial Registration:** https://www.clinicaltrials.gov/ct2/results?cond=&term=NCT04503629&cntry=&state=&city=&dist=, Identifier: CTR20181464, NCT04503629.

## What is Known

Duodenal ulcer (DU) is a common acid-related gastrointestinal disorder.

Proton pump inhibitors (PPIs) are validated gastric acid suppressors. PPIs have been widely used to treat patients with active duodenal ulcers.

Anaprazole {(R)-2-[[[4-(3-methoxylpropoxy)-3-methyl-2-pyridyl]methyl]sulfiny]-6,7-dihydro-3H-benzofuro [5,6-d] imidazole sodium}, a newly developed PPI with new chemical structure.

## What is New Here

Anaprazole, a novel PPI, can be used to treat patients with active duodenal ulcers effectively.

Both at a dosage of 20 and 40 mg daily, anaprazole, are effective and have good safety profile in the treatment of active duodenal ulcers.

Potential possible better liver tolerance was observed in two anaprazole dose groups than rabeprazole 10 mg group.

## Introduction

Duodenal ulcer (DU) is a common acid-related gastrointestinal disorder that has a high incidence in clinical practice in China. Consequently, gastric acid suppression has been widely accepted as the main treatment strategy for DUs worldwide. Comparative studies have demonstrated that proton pump inhibitors (PPIs) can provide better improvement of acid suppression, ulcer healing and pain relief than histamine 2 receptor antagonists (H2RAs) (1).

Previous preclinical trials showed that rabeprazole, a commonly used PPI in DU patients that has shown great curative efficacy in terms of promoting ulcer healing and clinical symptom relief, is superior to other PPIs, such as omeprazole, lansoprazole and pantoprazole, in the inhibition of H^+^/K^+^-ATPase (2– 4). Although 7-day dose-ranging studies have verified that rabeprazole 20 mg daily is more likely to provide an optimal gastric acid inhibitory effect, particularly in patients with peptic ulcer (5, 6), 10 mg rabeprazole per day is still the most extensively used dosage in China for individuals with peptic ulcer diseases. Although existing PPIs have shown great efficacy, there are clinical needs for PPIs with better safety profile in case of various adverse reaction of PPIs. Many scientists are devoted to developing more effective PPIs with better safety profile. Anaprazole is developed from these needs.

Anaprazole {(R)-2-[[[4-(3-methoxylpropoxy)-3-methyl-2-pyridyl]methyl]sulfiny]-6,7-dihydro-3H-benzofuro [5,6-d] imidazole sodium}, a newly developed PPI with new chemical structure. In preclinical and phase I clinical trials, anaprazole has shown an equivalent half-life and pharmacodynamics to that of rabeprazole in the suppression of gastric acid secretion but has demonstrated better safety profiles with lower incidence rate of gastrointestinal adverse reaction (7). A randomized, double-blinded, placebo parallel controlled phase 1 study of multiple ascending dose administration of anaprazole in healthy Chinese subjects has investigated pharmacodynamics data (the time percentage of gastric pH value > 3 and pH value > 4 in 24 h gastric pH monitoring post dose at day 6). Anaprazole 20 mg (20 mg, qd, 6 days) shows 61.16% (pH > 3) and 49.61% (pH > 4). Anaprazole 40 mg (40 mg, qd, 6 days) shows 71.29% (pH > 3) and 62.24% (pH > 4). In addition, anaprazole has less influence of CYP2C19 genotypes. Anaprazole is metabolized *via* systemic non-enzymatic reduction and multiple cytochromes P450 in the liver. In multiple kinds of cytochromes P450, CYP2C19 only contributes 6.88%. *In vitro* drug-drug interaction studies of anaprazole demonstrated anaprazole has very low risk of drug-drug interaction, especially for interaction on other drugs. To further evaluate the efficacy and safety of anaprazole, we conducted this multicenter, randomized, positive-controlled, double-blinded phase II clinical study mainly to address the following two objectives: (1) to evaluate the therapeutic efficacy and safety of anaprazole in patients with active DUs compared with rabeprazole and (2) to explore the optimal dose of anaprazole (20 vs. 40 mg).

## Methods

### Study Design

This multicenter, randomized, positive-controlled, double-blinded, parallel-group clinical study was conducted at 18 tertiary hospitals in China between October 2018 and April 2019. The study protocol was reviewed and approved by the NMPA of China (clinical trial ID: 2018L02415) and the Ethics Committee and institutional review board of each participating center. The study was carried out in accordance with the principles of the Declaration of Helsinki. A total of 150 patients with active DU were recruited. All participants signed written informed consent forms prior to enrollment.

### Patients

Patients were eligible for inclusion in this study if they met the following criteria: (1) age>18 years; (2) endoscopic diagnosis of active DU (stage of ulcer: A1 or A2) within 7 days prior to enrollment; (3) the presence of 1 or 2 ulcers with a larger diameter between 3 and 15 mm; and (4) willingness to sign the informed consent form.

The exclusion criteria were as follows: (1) patients with stress ulcers, complex ulcers, malignant ulcers or ulcers with cancerization risk; (2) patients who had esophageal erosion/ulcer, reflux esophagitis, varices of esophageal/fundus of stomach, Zollinger-Ellison syndrome; (3) patients who had severe complications, such as pyloric obstruction, active bleeding, or perforation; (4) patients with other severe gastrointestinal diseases such as active gastric ulcer or inflammatory bowel diseases; (5) patients with a history of upper gastrointestinal surgery to remove esophageal/stomach/duodenal tissue; (6) patients who failed to undergo complete endoscopies; (7) pregnant patients or those who were breastfeeding or preparing for pregnancy; (8) patients who had taken PPIs within 5 days or for over 3 consecutive days within 2 weeks prior to enrollment; (9) patients who underwent PPI-based triple/quadruple therapy for *Helicobacter pylori* (*H. pylori*) eradication within 28 days prior to enrollment; or (10) patients with consecutive use of medications inducing ulcer bleeding (e.g., steroids, non-steroidal anti-inflammatory drugs (NSAIDs), anticoagulants, or antiplatelets therapy) for > 3 days within 28 days prior to enrollment.

After the screening period, recruited patients (*n* = 150) were randomly assigned to 1 of the 3 following treatment groups, at a 1:1:1 ratio in accordance with a randomization list generated by a central interactive web response system (IWRS): (1) the 10 mg rabeprazole sodium enteric-coated tablets group, wherein patients received a 10-mg rabeprazole tablet (Eisai Pharmaceutical Company Ltd, Tokyo, Japan) together with 1 simulant placebo of 40 mg anaprazole and 1 simulant placebo of 20 mg anaprazole; (2) the 20 mg anaprazole sodium enteric-coated tablets group, wherein patients received a 20-mg anaprazole tablet (Sihuan Pharmaceutical Company Ltd, Beijing, China) together with one simulant placebo of 40 mg anaprazole and one simulant placebo of 10 mg rabeprazole; or (3) the 40 mg anaprazole sodium enteric-coated tablets group, wherein patients received a 40-mg anaprazole tablet (Sihuan Pharmaceutical Company Ltd, Beijing, China) together with 1 simulant placebo of 20 mg anaprazole and 1 simulant placebo of 10 mg rabeprazole. For the sake of blinding, placebos were identical in appearance, color and flavor to the two study drugs. All medications were orally administered once daily 30–60 min before breakfast for 4 consecutive weeks. *H. pylori* infections were diagnosed by the 13C-urea breath test and endoscopic examination with biopsy of the gastric mucosa.

### Efficacy Assessment

Efficacy evaluation of endoscopic findings and clinical symptoms was performed during this study. The primary endpoint was the ulcer healing rate at week 4 as assessed endoscopically by investigator review from each tertiary hospital. In addition, considering many investigators participated in this study and the evaluation of ulcer healing stage is usually subjective, endoscopic ulcer healing was assessed by blinded independent central review, which was applied to minimize assessment biases and variability. The independent central review committee consisted of three members: one chairman and two members. Two members independently provided specific assessment reports on the endoscopic findings. If these two assessment reports were inconsistent, the chairman and two members reached an agreement through consultation. If consensus could not be reached, the Chairman of the Committee made the final decision. This kind of independent review is widely recommended by many national official organizations to be used in many oncology clinical trials with endpoint of image.

The stage of the ulcer was endoscopically evaluated on the basis of the degree of ulceration, regenerating epithelialization and scarring, as demonstrated by previous studies (8, 9) as follows: A (active, A1 & A2) stage, where A1 stage is more severe than A2 stage; H (healing, H1 & H2) stage, in which H2 stage was better than H1 stage; and S [scarring, S1 (red scar) & S2 (white scar)] stage, where S2 stage was better than S1 stage. “Healing” of the ulcer was defined as an ulcer that was downstaged from A (A1 or A2) stage to S (either S1 or S2) stage, and if two ulcers were found, a healing state was determined only if all the ulcers had resolved to S stage. The healing rate of the ulcer is described as the percentage of the number of patients who achieved a healing state (endoscopic S stage) among all patients in each group. If adverse events occurred during the study period, it the investigators determined whether or not the patients could continue in the study.

The secondary endpoint included the time to achievement of complete epigastric pain relief and nocturnal epigastric pain relief and the percentage of patients who were free from epigastric pain or nocturnal epigastric pain at week 2 and week 4. Complete relief of epigastric pain was defined as the disappearance of epigastric pain without recurrence.

### Safety Evaluation and Drug Compliance Analysis

Safety was assessed on the basis of adverse events and common safety indexes at each visit. Adverse events were monitored throughout the whole study, including treatment-emergent adverse events (TEAEs), drug-related TEAEs, drug-related treatment-emergent serious adverse events (TESAEs), TEAEs leading to drug discontinuation, TEAEs leading to withdrawal from the study and TEAEs leading to death. Common safety indexes, including but not limited to vital signs, physical examination, routine laboratory examinations and electrocardiography, were obtained at the start and end of the study. Drug compliance within the acceptable range of 80–120%, actual medication in total (mean ± SD, tablet/capsule), drug exposure time (mean ± SD, day), and treatment frequency (mean ± SD, tablet/capsule per day) were evaluated.

### Statistical Analysis

Based on previous studies (10–12), the 4-weeks endoscopic duodenal ulcer healing rate was assumed to be 90% for rabeprazole. As a novel PPI, anaprazole will be taken to be effective only if the lower bound of 95% confidence interval (95% CI) of 4-weeks ulcer healing rate difference between anaprazole and rabeprazole is larger than −20% from the previous studies (13, 14) for comparing PPI and H2-Receptor Inhibitors. The 4-weeks endoscopic duodenal ulcer healing rate was assumed to be 90% for both anaprazole and rabeprazole. Considering a non-inferiority margin of−20%, and a drop-out rate near 20%, with type I error rate of 0.05, 50 patients in each group will provide a power of 85% to detect a non-inferiority result if there is one.

A full analysis set (FAS), per-protocol set (PPS), and safety analysis set (SS) were used for analysis in this study. The FAS population included all patients with a baseline evaluation and at least one dose of the study drug and post-treatment efficacy assessment, whereas the PPS population referred to those in the FAS population who had successfully finished the whole study without the absence of primary endpoint evaluation and major protocol deviation. The SS population included all patients who received at least one dose of study drug and had at least one follow-up safety evaluation data, which was only used for safety analysis and drug compliance analysis.

Demographic characteristics and other clinical baseline data are presented with descriptive statistics and were analyzed by ANOVA, a non-parametric test or the χ^2^ test to analyze the differences in the baseline characteristics between groups. The healing rates of the groups were assessed with the 95% confidence interval (95% CI) using the Farrington-Manning method. Multivariate logistic regression analysis was applied in the FAS and PPS populations to identify the risk factors related to ulcer healing based on *H. pylori* status (positive vs. negative), the number of ulcers (1 vs. 2), sex (male vs. female) and the 3 different treatments, all of which were either reported as ulcer healing-related risk factors or requested by the official center of drug evaluation from the point of pharmacological view (15, 16). Then, stratification analysis was carried out according to the results of the multivariate logistic regression analysis. Clinical symptom relief rates, such as epigastric pain and nocturnal epigastric pain, were evaluated by the Kaplan–Meier method. Descriptive statistics were used to analyze adverse events, vital signs, physical examination, electrocardiography, and routine laboratory examinations. All statistical analyses were performed using the SAS 8.2 software package, and a *P*-value < 0.05 was considered statistically significant.

## Results

### Demographic Characteristics and Compliance Analysis

**Figure 1:**
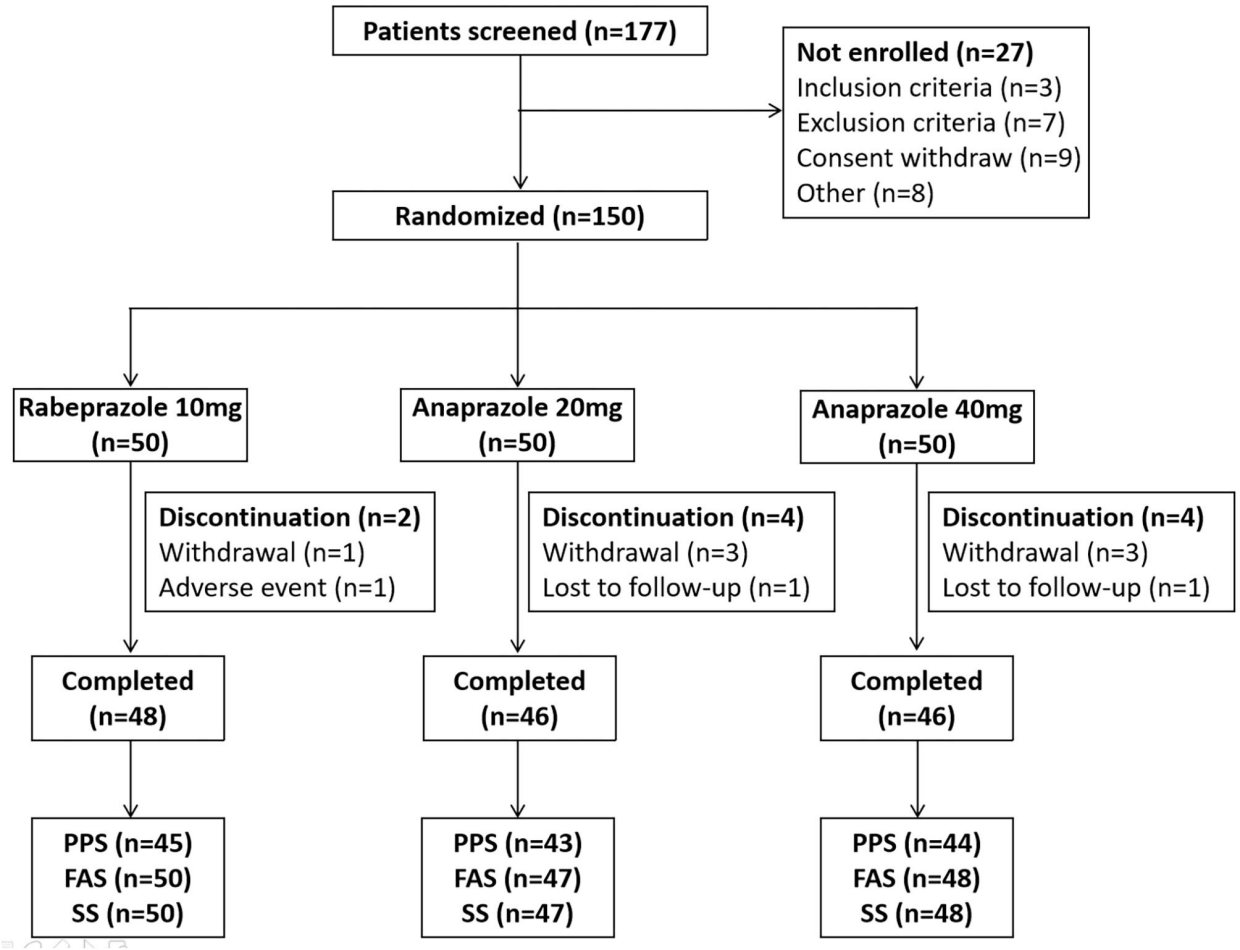
Flow diagram of the participants in this study. PPS, per-protocol set; FAS, full analysis set; SS, safety analysis set.

A total of 177 patients were screened, and 27 patients were excluded because they did not meet the inclusion or exclusion criteria, because they withdrew consent, or for other reasons. In total, 150 patients who met the inclusion criteria were included in this study and assigned randomly to the three treatment groups (50 patients in the 10 mg rabeprazole group, 50 patients in the 20 mg anaprazole group and 50 patients in the 40 mg anaprazole group). Of these patients, 145 patients received at least one dose of the study drug, and 140 patients completed the study. During the study, a total of 10 patients discontinued the drug primarily due to adverse events, withdrew consent or were lost to follow-up (Figure 1). The demographic and baseline characteristics of the patients involved in the FAS analysis were generally balanced between the treatment groups (Table 1). The rates of compliance within the acceptable range of 80–120% were 100, 100, and 97.9% for the 10 mg rabeprazole group, 20 mg anaprazole group and 40 mg anaprazole group, respectively (Table 1).

**Table 1:** Demographic characteristics and compliance in the FAS population.

**Parameter**	**Rabeprazole**	**Anaprazole**		***P*-value**
	**10 mg (*n* = 50)**	**20 mg (*n* = 47)**	**40 mg (*n* = 48)**	
**Demographic and clinical characteristics**				
Age (mean ± SD, y)	45.1 ± 10.75	39.5 ± 12.72	41.0 ± 11.97	0.0549
Males [No. (%)]	37 (74.0)	33 (70.2)	32 (66.7)	0.7299
Height (mean ± SD, cm)	167.83 ± 7.888	166.47 ± 8.418	167.06 ± 8.156	0.7117
Weight(mean ± SD, kg)	65.70 ± 12.251	62.64 ± 10.518	63.34 ± 9.540	0.3455
BMI (mean ± SD, kg/m^2^)	23.17 ± 3.456	22.49 ± 3.031	22.60 ± 2.562	0.4934
PU history [No. (%)]	25 (50.0)	21 (44.7)	22 (45.8)	0.8575
Other digestive disease [No. (%)]	41 (82.0)	37 (78.7)	37 (77.1)	0.8294
PU-related drug use history over 4 weeks [No. (%)]	7 (14.0)	5 (10.6)	11 (22.9)	0.2481
PU-related surgery history [No. (%)]	0	1 (2.1)	1 (2.1)	0.9982
*H. pylori* status, positive [No. (%)]	40 (80.0)	37 (78.7)	38 (79.2)	0.9876
**Endoscopic findings**				
Number of ulcers				
1	36 (72.0)	38 (80.9)	39 (81.3)	0.4613
2	14 (28.0)	9 (19.1)	9 (18.8)	0.4613
Location of ulcers: duodenal bulb	49 (98.0)	47 (100.0)	48 (100.0)	0.9959
Size of ulcer 1 (mean ± SD) (mm)	7.4 ± 3.08	6.5 ± 2.76	6.4 ± 2.31	0.1214
Stage of ulcer 1 [No. (%)]				
A1	35 (70.0)	37 (78.7)	38 (79.2)	0.4910
A2	15 (30.0)	10 (21.3)	10 (20.8)	0.4910
Size of ulcer 2 (mean ± SD) (mm)	6.6 ± 3.33	6.2 ± 3.19	7.3 ± 2.29	0.7331
Stage of ulcer 2 [No. (%)]				
A1	9 (64.3)	7 (77.8)	7 (77.8)	0.7050
A2	4 (28.6)	2 (22.2)	2 (22.2)	0.9191
H1	1 (7.1)	0	0	0.9967
**Drug compliance**				
Compliance within the acceptable range of 80–120% [No. (%)]	50 (100%)	47 (100%)	47 (97.9%)	0.9958
Actual medication in total (mean ± SD, tablet/capsule)	27.2 ± 4.03	27.4 ± 3.95	26.6 ± 5.20	0.4930
Drug exposure time (mean ± SD, day)	27.3 ± 4.03	27.4 ± 3.95	27.1 ± 4.93	0.7590
Treatment frequency (mean ± SD, tablet/capsule per day)	0.994 ± 0.023	0.999 ± 0.011	0.984 ± 0.068	0.3684

*FAS, full analysis set; PU, peptic ulcer*.

### Evaluation of Ulcer Healing

The endoscopic ulcer healing rate assessed by investigators and independent central review is shown in Table 2. The ulcer healing rates of each treatment group in the FAS population at week 4 of follow-up were as follows: 88.0% in the 10 mg rabeprazole group, 85.1% in the 20 mg anaprazole group and 87.5% in the 40 mg anaprazole group by independent central review and 72.0% in the 10 mg rabeprazole group, 70.2% in the 20 mg anaprazole group and 77.1% in 40 mg anaprazole group by investigator review. By independent central review, the ulcer healing rate difference between anaprazole 20 mg and Rabeprazole 10 mg is −2.9% (95% CI, −16.5–10.7%), and −0.5% (95% CI, −13.5–12.5%) between anaprazole 40 mg and Rabeprazole 10 mg. By investigator review, the ulcer healing rate difference between anaprazole 20 mg and Rabeprazole 10 mg is −1.8% (95% CI, −19.8–16.3%), and 5.1% (95% CI, −12.2–22.3%) between anaprazole 40 mg and Rabeprazole 10 mg (Table 2). The ulcer healing rates of each treatment group in the PPS population at week 4 of follow-up were as follows: 88.9% in the 10 mg rabeprazole group, 86.0% in the 20 mg anaprazole group and 90.9% in the 40 mg anaprazole group by independent central review and 75.6% in the 10 mg rabeprazole group, 72.1% in the 20 mg anaprazole group and 79.5% in the 40 mg anaprazole group by investigator review. By independent central review, the ulcer healing rate difference between anaprazole 20 mg and Rabeprazole 10 mg is−2.8% (95% CI, −16.7–11%), and 2.0% (95% CI, −10.5–14.5%) between anaprazole 40 mg and Rabeprazole 10 mg. By investigator review, the ulcer healing rate difference between anaprazole 20 mg and Rabeprazole 10 mg is −3.5% (95% CI, −21.8–14.9%), and 4.0% (95% CI, −13.4–21.3%) between anaprazole 40 mg and Rabeprazole 10 mg (Table 2).

**Table 2:** Healing rates of duodenal ulcers up to week 4 in the FAS and PPS populations.

**Treatment**	**FAS population**		**PPS population**	
	**Independent central review**	**Investigator review**	**Independent central review**	**Investigator review**
**Healing rates**				
Rabeprazole 10 mg [No.] [(%) (95% CI)]	44 [88.0 (79.0–97.0)]	36 [72.0(59.6–84.5)]	40 [88.9(79.7–99.4)]	34 [75.6(63.0–88.1)]
Anaprazole 20 mg [No. ] [(%) (95% CI)]	40 [85.1(74.9–95.3)]	33 [70.2(57.1–83.3)]	37 [86.0(75.7–96.4)]	31 [72.1(58.7–85.5)]
Anaprazole 40 mg [No. ] [(%) (95% CI)]	42 [87.5(78.1–96.9)]	37 [77.1(65.2–89.0)]	40 [90.9(82.4–99.4)]	35 [79.5(67.6–91.5)]
**Difference in healing rate between groups**				
Anaprazole 20 mg-Rabeprazole 10 mg [% (95% CI)]	−2.9 (−16.5–10.7)	−1.8 (−19.8–16.3)	−2.8 (−16.7–11.0)	−3.5 (−21.8–14.9)
Anaprazole 40 mg-Rabeprazole 10 mg [% (95% CI)]	−0.5 (−13.5–12.5)	5.1 (−12.2–22.3)	2.0 (−10.5–14.5)	4.0 (−13.4–21.3)
Anaprazole 20 mg-Anaprazole 40 mg [% (95% CI)]	2.4 (−11.4–16.2)	6.9 (−10.8–24.6)	4.9 (−8.5–18.3)	7.5 (−10.5–25.4)

*FAS, full analysis set; PPS, per-protocol set; CI, confidence interval. Farrington-Manning analysis was used to assess the differences in healing rates between groups with the 95% confidence interval (95% CI)*.

### Logistic Regression Analysis and Subanalysis of Ulcer Healing

A logistic regression model that included *H. pylori* status, ulcer numbers, sex and different treatments as independent variables was conducted to identify the ulcer healing-related risk factors in the FAS and PPS populations. The results showed that *H. pylori* status [*p* = 0.031, OR = 0.273, 95% CI (0.084–0.891)] was an independent ulcer healing-related risk factor in the PPS population (Table 3). Therefore, subanalyses were further performed based on *H. pylori* status in the FAS and PPS populations (Table 4). The ulcer healing rates of the *H. pylori*-positive patients in the 10 mg rabeprazole, 20 mg anaprazole and 40 mg anaprazole groups were 90% (36/40), 86.5% (32/37) and 92.1% (35/38), respectively, in the FAS population and 91.7% (33/36), 88.6% (31/35) and 94.4% (34/36), respectively, in the PPS population. The ulcer healing rates of the *H. pylori*-negative patients in the 10 mg rabeprazole, 20 mg anaprazole and 40 mg anaprazole groups were 80.0% (8/10), 80.0% (8/10) and 70.0% (7/10), respectively, in the FAS population and 77.8% (7/9), 75.0% (6/8) and 75.0% (6/8), respectively, in the PPS population (Table 4).

**Table 3:** Multivariate regression analysis for risk factors related to ulcer healing.

	**Variables**	**FAS population**			**PPS population**		
		**Wald χ^2^**	***P***	**OR (95% CI)**	**Wald χ^2^**	***P***	**OR (95% CI)**
Therapy	20 mg ANA vs. 10 mg RAB	0.154	0.695	0.788 (0.240–2.588)	0.234	0.629	0.726 (0.198–2.658)
	40 mg ANA vs. 10 mg RAB	0.003	0.958	0.967 (0.284–3.295)	0.093	0.760	1.247 (0.302–5.158)
*H. pylori* status	Negative vs. positive	3.307	0.069	0.380 (0.134–1.078)	4.628	0.031	0.273 (0.084–0.891)
Number of ulcers	1 vs. 2	0.002	0.966	1.026 (0.308–3.418)	0.556	0.456	1.628 (0.452–5.863)
Sex	Male vs. female	0.067	0.795	1.150 (0.399–3.314)	0.350	0.554	0.663 (0.170–2.586)

*ANA, Anaprazole; RAB, Rabeprazole; FAS, Full analysis set; PPS, per-protocol set*.

**Table 4:** Subanalysis of ulcer healing rates by *H. pylori* in the FAS and PPS populations.

**Treatment**	**FAS population**		**PPS population**	
	**Hp- [No. (%)] Healed unhealed**	**Hp+ [No. (%)] Healed unhealed**	**Hp- [No. (%)] Healed unhealed**	**Hp+ [No. (%)] Healed unhealed**
Rabeprazole 10 mg	8 (80.0) 2 (20.0)	36 (90.0) 4 (10.0)	7 (77.8) 2 (22.2)	33 (91.7) 3 (8.3)
Anaprazole 20 mg	8 (80.0) 2 (20.0)	32 (86.5) 5 (13.5)	6 (75.0) 2 (25.0)	31 (88.6) 4 (11.4)
Anaprazole 40 mg	7 (70.0) 3 (30.0)	35 (92.1) 3 (7.9)	6 (75.0) 2 (25.0)	34 (94.4) 2 (5.6)
Total	23 (76.7) 7 (23.3)	103 (89.6) 12 (10.4)	19 (76.0) 6 (24.0)	98 (91.6) 9 (8.4)

*FAS, full analysis set; PPS, per-protocol analysis set. Hp, H. pylori*.

### Evaluation of Clinical Symptoms

**Figure 2:**
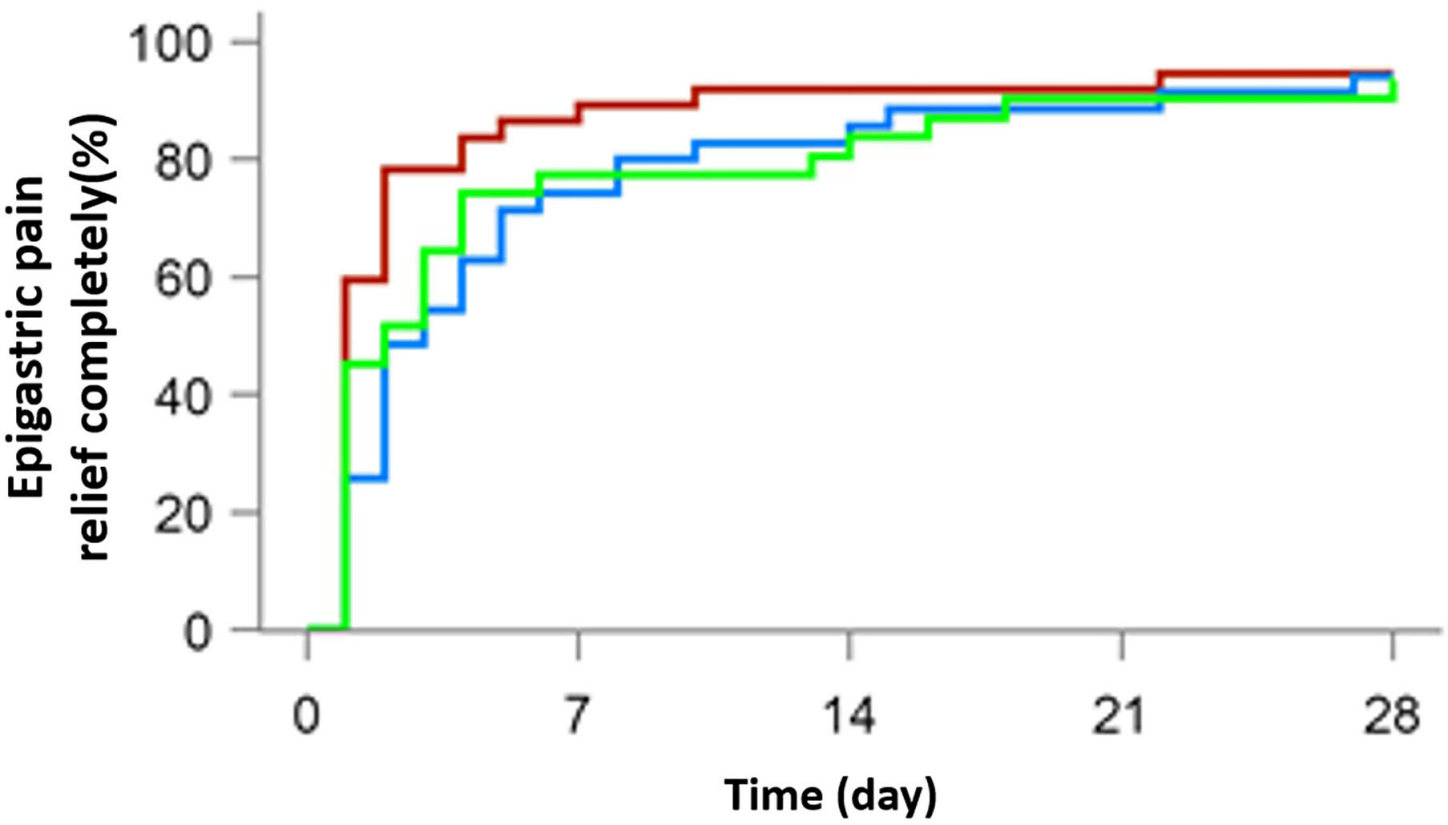
Cumulative percentages of patients with complete epigastric pain relief on days 7, 14, 21, and 28 of therapy with 10 mg rabeprazole (red line), 20 mg anaprazole (blue line), and 40 mg anaprazole (green line) in the FAS population.

**Figure 3:**
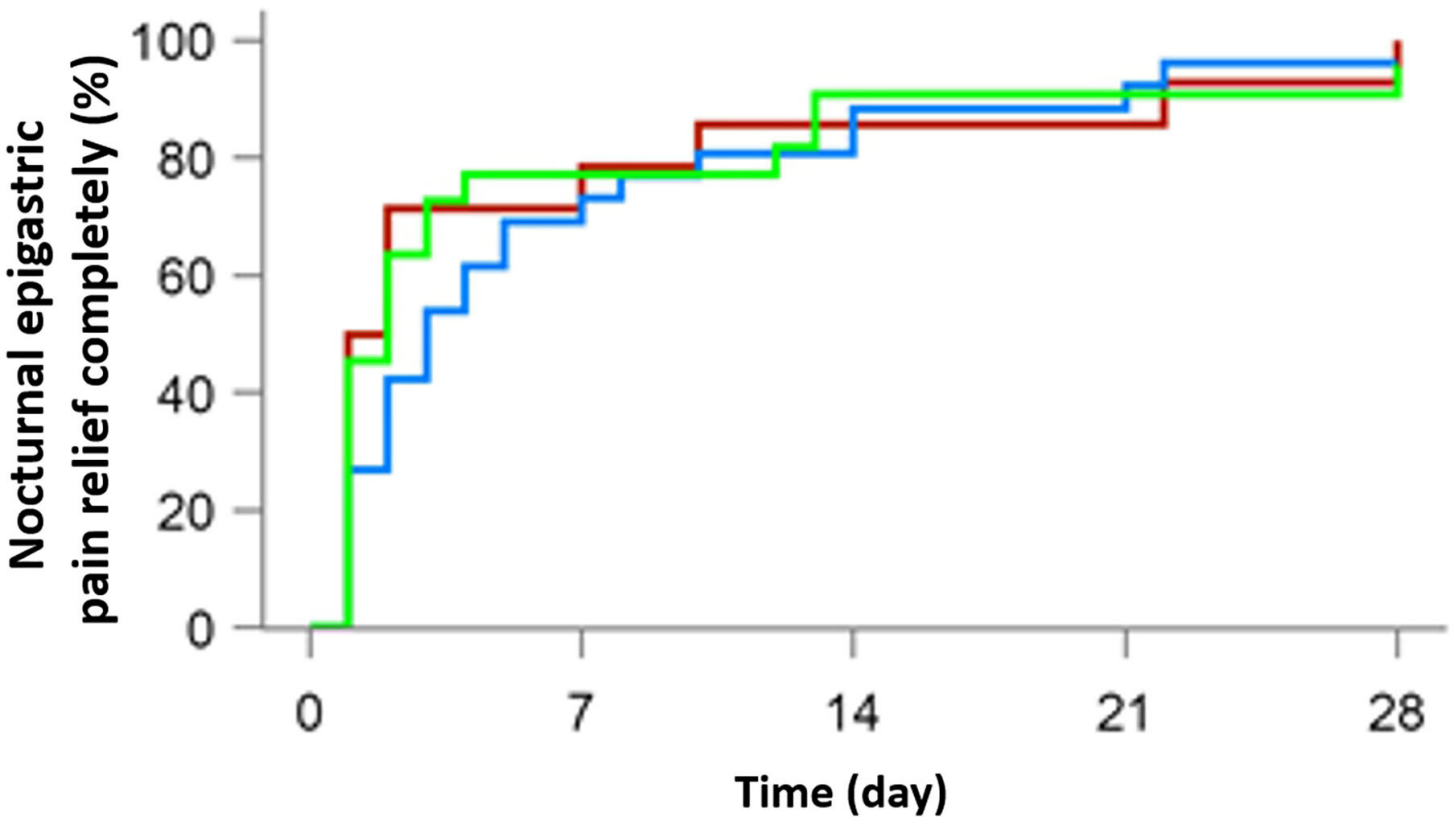
Cumulative percentage of patients with complete nocturnal epigastric pain relief on days 7, 14, 21, and 28 of therapy with 10 mg rabeprazole (red line), 20 mg anaprazole (blue line) and 40 mg anaprazole (green line) in the FAS population.

A total of 103 patients were suffering from epigastric pain (37 in the 10 mg rabeprazole group, 35 in the 20 mg anaprazole group and 31 in the 40 mg anaprazole group), while 62 had nocturnal epigastric pain (14 in the 10 mg rabeprazole group, 26 in the 20 mg anaprazole group and 22 in the 40 mg anaprazole group). Most people achieved complete symptom relief in weeks 2 and 4. After 4 weeks of treatment, 97 of 103 (94.2%) patients were free from epigastric pain, with 35/37 (94.6%) in the 10 mg rabeprazole group, 33/35 (94.3%) in the 20 mg anaprazole group and 29/31 (93.5%) in the 40 mg anaprazole group. Additionally, 60 of 62 (96.8%) subjects had complete nocturnal epigastric pain relief, with 14/14 (100%) in the 10 mg rabeprazole group, 25/26 (96.2%) in the 20 mg anaprazole group and 21/22 (95.5%) in the 40 mg anaprazole group. The median time to complete nocturnal epigastric pain relief was 1.5 days (95% CI: 1–7 days) in the 10 mg rabeprazole group, 3 days (95% CI: 2–5 days) in the 20 mg anaprazole group and 2 days (95% CI: 1–3 days) in the 40 mg anaprazole group. Additionally, the Kaplan-Meier curves of the three treatment groups were extremely consistent (Figures 2, 3).

### Safety Assessments

Safety analysis was performed in the SS population during the study period (*n* = 145). A total of 81 adverse events were recorded among 47 of 145 (32.4%) patients, with 17 of 50 (34.0%) patients in the 10 mg rabeprazole group, 16 of 47 (34.0%) patients in the 20 mg anaprazole group and 14 of 48 (29.2%) patients in the 40 mg anaprazole group. The incidences of adverse events were similar among the three groups. Only one patient withdrew from the study due to TESAEs (not drug-related) in the 10 mg rabeprazole group, while no patient withdrew from the study in the other two treatment groups.

Of the 47 patients with adverse events during this study, 24 (16.6%) patients experienced drug-related (definitely, probably, or possibly) TEAEs, whereas none of these events were severe, with 9 of 50 (18%) patients in the 10 mg rabeprazole group and 9 of 47 (19.1%) and 6 of 48 (12.5%) patients in the 20 and 40 mg anaprazole groups, respectively. The incidences of drug-related TEAEs of the three treatments were of no significant difference. Gastrointestinal disorders were the most commonly reported adverse events in the three treatment groups. The incidences of gastrointestinal disorders were 10.0% (5/50) in the 10 mg rabeprazole group, 12.8% (6/47) in the 20 mg anaprazole group and 14.6% (7/48) in the 40 mg anaprazole group. There were 6 of 50 (12%) patients with liver dysfunction TEAEs (3 patients with drug-related TEAEs) in the 10 mg rabeprazole group, but both 20 mg and 40 mg anaprazole group had no adverse events related to liver dysfunction (Table 5).

**Table 5:** TEAEs in patients in the safety analysis set.

	**Rabeprazole**		**Anaprazole**			
	**10 mg (*****n*** **= 50)**		**20 mg (*****n*** **= 47)**		**40 mg (*****n*** **= 48)**	
	**Patients (%)**	**Events**	**Patients (%)**	**Events**	**Patients (%)**	**Events**
Any TEAE	17 (34.0)	39	16 (34.0)	22	14 (29.2)	20
Mild	17 (34.0)	35	14 (29.8)	20	14 (29.2)	18
Moderate	4 (8.0)	4	2 (4.3)	2	1 (2.1)	2
Severe	0	0	0	0	0	0
Drug-related TEAEs	9 (18.0)	16	9 (19.1)	9	6 (12.5)	7
Drug-related SAEs	0	0	0	0	0	0
TESAEs (not drug-related) leading to drug discontinuation and withdrawal	1 (2.0)	1	0	0	0	0
TEAE leading to death	0	0	0	0	0	0
**Frequently reported TEAEs**						
Gastrointestinal disorders	5 (10.0)	6	6 (12.8)	6	7 (14.6)	9
Abdominal distension	2 (4.0)	2	0	0	1 (2.1)	1
Nervous system disorders	5 (10.0)	7	2 (4.3)	2	1 (2.1)	1
Dizziness	2 (4.0)	2	1 (2.1)	1	0	0
Headache	2 (4.0)	4	0	0	0	0
Liver dysfunction	6 (12.0)	7	0	0	0	0
Infections and infestation	2 (4.0)	2	2 (4.3)	2	2 (4.2)	2
Upper respiratory infection	0	0	1 (2.1)	1	1 (2.1)	1

*SAE, serious adverse event; TEAEs, treatment-emergent adverse events; TESAEs, treatment-emergent serious adverse events*.

## Discussion

As an acid-related disease, DUs are quite common, and the relationship between gastric acid inhibition and healing of ulcers has been clearly noted in the treatment of DU (17). Among gatric acid inhibitory medications, PPIs are the most efficient pharmacological agents in reducing gastric acid secretion and are currently the mainstay of medical therapy for peptic ulcers (18). Among all the PPIs, rabeprazole is frequently used in clinical practice and has been verified to be a more potent repressor of H^+^/K^+^-ATPase than many other PPIs and to ensure longer-lasting acid suppression and better safety performance (10, 19, 20). Moreover, unlike other PPIs, rabeprazole is metabolized to the thioether compound *via* a non-enzymatic pathway with minor involvement of cytochrome P450 (CYP) 2C19 (11, 12), which is similar to anaprazole according to our preliminary study (7). Hence, we selected rabeprazole as the positive control medication in this clinical study to comparatively assess the healing efficacy of anaprazole.

Anaprazole is a novel PPI with new chemical structure. New structure gives feature to Anaprazole. Anaprazole is metabolized *via* non-enzyme and multiple kinds of cytochrome P-450 enzyme. CYP2C19 only contributes 6.88%, therefore gene polymorphism has very less impact on Anaprazole. *In vitro* studies (undisclosed data) in cytochrome P-450 enzyme and transporter show very small risk of drug-drug interaction. Since anaprazole is a novel PPI, this is the first study to evaluate the ulcer healing efficacy of anaprazole in patients with active DUs. In this phase II clinical study, the DU healing rates were 88.0% in the 10 mg rabeprazole group, 85.1% in the 20 mg anaprazole group and 87.5% in the 40 mg anaprazole group in the FAS population, and 88.9% in the 10 mg rabeprazole group, 86.0% in the 20 mg anaprazole group and 90.9% in the 40 mg anaprazole group in the PPS population by independent central review. The results revealed that all treatments had a high ulcer healing rate at the 4-week follow-up. The lower bound of two-sided 95% CI of ulcer healing difference (anaprazole 20 mg-Rabeprazole 10 mg; anaprazole 40 mg-Rabeprazole 10 mg) is larger than −20%, which shows both anaprazole 20 and 40 mg is effective in treatment of active duodenal ulcers. This result allows both anaprazole 20 and 40 mg to be advanced to a phase III clinical trial. The ulcer healing rates by investigator review could lead to the same conclusion. However, the ulcer healing rates by investigator review were approximately 10% lower than those by independent central review, which may be the result of assessment biases and variability.

Numerous factors, such as *H. pylori* infection, ulcer numbers, ulcer site and other comorbidities, may influence the healing of DUs. As documented in a previous study (21), the average *H. pylori* positivity rate has reached approximately 50% in China, and in most cases, active DU patients are coinfected with *H. pylori*. Previous studies indicated that *H. pylori* eradication reduced the complication incidence and relapse rate of peptic ulcers. In addition, Labenz et al. (22) reported that *H. pylori* correlated with ulcer healing in PPI treatment. Similarly, our study on risk factors for ulcer healing showed that *H. pylori* infection may be an independent protective factor for DU healing by multivariate logistic regression analysis, especially in the PPS population (*P* = 0.031; OR = 0.273, 95%CI = 0.084–0.891). According to the subanalysis, the DU healing rates of *H. pylori*-positive patients were 89.6% in the FAS population and 91.6% in the PPS population, and the healing rates of *H. pylori*-negative patients were 76.7% in the FAS population and 76.0% in the PPS population (Table 4). This study didn't exclude H.pylori negative patients in screening period. The percentage of H.pylori negative subjects is similar among anaprazole 20 mg group (21.3%), anaprazole 40 mg group (20.8%) and rabeprazole 10 mg group (20%). H.pylori positive subjects accounted for 80% or so and received the recommended eradication therapy after completion of the study, same as other studies (23). The demographic and baseline characteristics of the patients involved in the FAS analysis were generally balanced between the treatment groups, which shows the factors had no impacts on the major conclusion in this study.

In the present study, a total of 103 patients suffered from epigastric pain, and 62 patients had nocturnal epigastric pain. As expected, the majority of patients who had clinical symptoms at baseline were ultimately asymptomatic after treatment, regardless of the treatments to which they were assigned. However, the improvement in the severity of epigastric pain was also similar in both groups, which was unrelated to *H. pylori* status, ulcer numbers or the sex of the patients. Although there were still a few patients who did not reach complete remission of symptoms, the degree of epigastric pain was considerably alleviated. In fact, it is not surprising to find some patients experiencing persistent epigastric pain after standard therapy (24). Moreover, patients without any symptoms at baseline sometimes present with mild symptoms after treatment, which may be due to other reasons, such as functional gastrointestinal disorders.

With respect to the safety and tolerance profile, only a small number of patients developed drug-related TEAEs, with 9 of 50 (18%) patients in the 10 mg rabeprazole group and 9 of 47 (19.1%) and 6 of 48 (12.5%) patients in the 20 mg and 40 mg anaprazole groups, respectively. The incidences of drug-related TEAEs of the three treatments were of no significant difference. According to previous studies, symptoms in the digestive, circulatory and nervous systems are the most common PPI-related adverse events (25–27). Similarly, gastrointestinal disorders were the most commonly reported adverse events in our study. Only one patient in the rabeprazole group discontinued treatment and withdrew from the study due to TESAEs (not drug-related). All of the adverse events were mild or moderate and resolved without any additional therapy. As a result, we concluded that all the medications were safe and well-tolerated in the three treatment groups. Both anaprazole 20 and 40 mg daily use of 4 weeks has good safety profile and tolerance. TEAEs related to liver dysfunction (liver enzyme increase, such as ALT or AST) happened in Rabeprazole and hadn't been reported in both 20 and 40 mg anaprazole group. Adverse reactions of liver enzyme increase were found in Rabeprazole's label and other PPIs' label. Liver enzyme increase after drug administration were taken as a signal of liver dysfunction or liver intolerance in Chinese clinical practice. ALT and AST had been tested at baseline before the drug administration in this study. These cases with liver enzyme increase were judged as liver dysfunction drug-related TEAEs by investigators based on PPIs label. From this perspective, this study shows anaprazole might have potential better liver tolerance than rabeprazole. This benefit of anaprazole need to be confirmed in larger sample size study.

Our study had several limitations. First, the sample size for subgroup analysis was relatively small, so the impact of variance was relatively large. As there are no explicit data exhibiting the efficacy of anaprazole in the treatment of active DUs in comparison with other PPIs at present, this phase II clinical study was performed as an exploratory study to lay the foundation for a subsequent corroborative phase III clinical study. Second, the follow-up duration was only 4 weeks, so information about ulcer relapse after 4 weeks was not collected. Accordingly, long-term studies with expanded sample sizes should be carried out in the future. Last, the patients in the study were all from China. It has been previously reported that there are many differences between DUs in the east and the west (28, 29). Thus, further studies are needed in Western countries.

In summary, we conclude that both 20 and 40mg anaprazole was evaluated to be effective, safe and tolerable. 20 mg is enough from cost-effectiveness and recommended in clinical practice. Next, a large-scale phase III clinical study will be conducted to further explore the therapeutic efficacy and safety of anaprazole in active DU treatment. Additionally, to obtain optimum benefits from anaprazole and observe how it can be applied in clinical practice, other studies targeting drug interaction, *H.pylori* eradication, will be carried out to investigate the characteristics that might differentiate anaprazole from other PPIs.

## Data Availability Statement

The original contributions presented in the study are included in the article/supplementary material, further inquiries can be directed to the corresponding author/s.

## Ethics Statement

The studies involving human participants were reviewed and approved by Ethics Committee and institutional review board of each participating center. The patients/participants provided their written informed consent to participate in this study. Written informed consent was obtained from the individual(s) for the publication of any potentially identifiable images or data included in this article.

## Author Contributions

NL, XH, and LH were involved in the study design and critical revision of the manuscript. XS, ZZ, and YF were involved in drafting of the manuscript. All authors were involved in the acquisition of data and interpretation of study results. All authors approved the final version of the manuscript, including the authorship list. Guarantor of the article: NL.

## Conflict of Interest

YC and XD are employed by XuanzhuBiopharmaceutical Co., Ltd., Beijing. The remaining authors declare that the research was conducted in the absence of any commercial or financial relationship that could be construed as a potential conflict of interest. The authors declare that this study received funding from Xuanzhu Biopharmaceutical Co., Ltd. The funder had the following involvement in the study: study design, data collection, and data analysis.
